# Prevalence, incidence, and risk factors of primary open-angle glaucoma - a cohort study based on longitudinal data from a German public health insurance

**DOI:** 10.1186/s12889-019-6935-6

**Published:** 2019-07-01

**Authors:** D. Kreft, G. Doblhammer, R. F. Guthoff, S. Frech

**Affiliations:** 1 Institute for Sociology and Demography, University of Rostock, Ulmenstrasse 69, 18057 Rostock, Germany; 2 Rostock Center for the Study of Demographic Change, Konrad-Zuse-Str. 1, 18057 Rostock, Germany; 3 German Center for Neurodegenerative Diseases (DZNE), Sigmund-Freud-Straße 27, 53105 Bonn, Germany; 4 Department of Ophthalmology, Rostock University Medical Center, Doberaner Str. 140, 18057 Rostock, Germany

**Keywords:** Glaucoma, Prevalence, Incidence, Risk factors, Cox model, Epidemiology, Diabetes, Health claims data, Validation

## Abstract

**Background:**

This study estimates the prevalence and incidence rates of primary open -angle glaucoma (POAG) as well as risk factors based on a dataset from the largest German health insurance company.

**Methods:**

A random sample of 250,000 persons at age 50+ of the Allgemeine Ortskrankenkasse (AOK) from 2010 to 2013 was used. Selected risk factors of POAG incidence were analyzed using multivariate Cox proportional hazard models.

**Results:**

The age-standardized prevalence of POAG at age 50+ in 2010 was 2.79% (95%-CI: 2.72–2.85%). The age-standardized total incidence rate was 0.38 (0.36–0.39) per 100 person-years. Sex differences were significant for total prevalence and total incidence rates, with higher prevalence and incidence rates for women compared to men. The Cox model revealed a strong age effect, a significantly 19% higher incidence for women (*p* ≤ 0.001), injuries of the eye and orbit (175%, *p* ≤ 0.001), degeneration of iris and ciliary body (155%, *p* = 0.022), myopia (155%, *p* ≤ 0.001), retinal vascular occlusions (134%, *p* ≤ 0.001), hypertension (13%, *p* ≤ 0.001) and diabetes mellitus (23%, *p* ≤ 0.001).

**Conclusion:**

Health claims data are an important data source for estimating POAG occurrence and help overcome the problems of small sample sizes. These results may help to understand the causal pathways of POAG and to develop intervention strategies to increase the awareness of patients and physicians with the aim of reducing POAG incidence.

**Electronic supplementary material:**

The online version of this article (10.1186/s12889-019-6935-6) contains supplementary material, which is available to authorized users.

## Background

Vision impairment is a major public health issue and population ageing will lead to an increasing burden over the next decades. Glaucoma, one of the leading causes of blindness, is a chronic optic neuropathy with irreversible but preventable visual field loss and progressive optic nerve damage [1]. It is generally asymptomatic until late in the disease, at which point permanent visual problems arise [2]. Therefore early detection and appropriate treatment is essential [3], which can be facilitated by better knowledge of the prevalence and incidence of glaucoma, and the risk factors associated with primary open-angle glaucoma (POAG), which is the most common type of glaucoma [1]. Hence, the aim of this study was twofold. First, we wanted to provide new epidemiologic information about POAG based on a large data set from a public health insurer and compared this with published data. Second, we wanted to explore selected disease-related risk factors of POAG incidence in an individual-level longitudinal design, as only limited information has been available thus far.

To put our results in the context of earlier studies, a systematic review with the keywords “glaucoma”, “prevalence”, and “incidence” based on Medline, distinguishing between total prevalence and age-specific prevalence and incidence, was performed.

In 2010, an estimated 44.7 million people worldwide suffered from primary open-angle glaucoma (POAG), and 4.5 million were blind, making POAG the most common type of glaucoma [4]. Recently, Kapetanakis et al. updated these numbers [5]. In 2015, 57.5 million people worldwide were affected by POAG and 7.8 million persons in Europe. Thus, the estimated total prevalence for Europe was 2% and the global prevalence was 2.2%. Cedrone et al. estimated a POAG prevalence of 2.51% for residents of Ponza, Italy [6]. In a meta-analysis by Tham et al. the total global prevalence of POAG was 3.54% for ages 40–80 [7]. For the European population, the total prevalence was 2.51% with a 36% higher prevalence in males than in females [7].

In total, 11 studies of age- and sex-specific estimates of the prevalence of POAG in European and European-derived populations were identified (Table 1) [5, 8–17]. Although the study populations were assumed to be homogenous between the studies, the variation of the estimated prevalence was comparatively high. Among these studies, the total prevalence varied between 0.8% [12] and 2.40% [9]. Friedman et al. stated a prevalence of 8.5% [14], but the population was restricted to persons aged 73+. All studies found a steep increase of the prevalence with age.

**Table 1:** 1 Age- and sex-specific prevalence of POAG from selected studies, only European and European-derived populations

		40–49		50–59		60–69		70–79		80+			Total
Tielsch et al. 1991 [8]	Definite POAG	0.92		0.41		0.88		2.89		2.16			1.29
Baltimore, USA	CI	0–2.72		0–0.98		0.14–1.62		1.44–4.34		0.05–4.26			0.80–1.78
White people													
					< 60	60–69		70–79		80+			Total
Mitchell et al. 1996 [9]	Definite POAG				0.30	1.10		4.20		8.20			2.40
Sydney, Australia													
		40–49		50–59		60–69		70–79		80–89		90+	Total
Wensor et al. 1998 [10]	Definite POAG	0.10		0.60		1.90		5.20		5.50		11.80	1.70
Melbourne, Australia													
		40–44	45–49	50–54	55–59	60–64	65–69	70–74	75–79	80–84	85–89	90+	Total
Tuck & Crick 1998 [11]	Fitted values	0.12	0.21	0.35	0.59	0.97	1.51	2.25	3.12	4.01	4.33		1.23
Pooled data, estimations	definite POAG												
					55–59	60–64	65–69	70–74	75–79	80+			Total
Wolfs et al. 2000 [12]	Men (Definite)				0.20	0.60	0.80	1.30	1.90	3.60			1.10
Rotterdam, The Netherlands	Women (Definite)				0.00	0.10	0.90	1.10	0.60	1.30			0.60
	Total (Definite)				0.10	0.40	0.90	1.20	1.10	2.00			0.80
		40–44	45–49	50–54	55–59	60–64	65–69	70–74	75–79	80–84	85–89	90+	Total
Tuck & Crick 2003 [13]	Fitted values	0.22	0.33	0.51	0.77	1.15	1.68	2.41	3.35	4.48	5.72	7.24	1.44
Pooled data, estimations	definite POAG												
		40–49		50–54	55–59	60–64	65–69	70–74	75–79	80+			
Friedmann et al. 2004 [14]	Men	0.83		0.89	1.02	1.23	1.58	2.16	3.12	6.94			
Pooled data from former studies	CI	0.65–1.06		0.78–1.02	0.89–1.16	1.07–1.41	1.37–1.82	1.87–2.49	2.68–3.63	5.4–8.88			
White people only	Women	0.36		0.61	0.85	1.18	1.64	2.27	3.14	5.58			
	CI	0.27–0.47		0.5–0.74	0.72–1	1.02–1.37	1.4–1.91	1.9–2.72	2.53–3.9	4.15–7.47			
								73–74	75–79	80–84	85+		Total
Friedmann et al. 2006 [15]	Total							3.40	9.30	7.30	13.10		8.50
Salisbury, USA	CI							0.5–6.4	6.5–12.2	3.9–10.6	7.7–18.4		6.7–10.3
White persons													
		40–49		50–59		60–69		70–79		80–89		90–95	
Rudnicka et al. 2006 [16]	Total	0.40		0.80		1.60		3.30		6.60		10.80	
Meta-Analysis, White people	CI	0.3–0.6		0.5–1.2		1.1–2.5		2.2–4.9		4.4–9.7		7.2–15.8	
in Europe, America, and Australia													
			< 49	50–59		60–69		70–79		80–89		90+	Total
Wolfram & Pfeiffer 2012 [17]	Raw		0.48	3.40		9.00		14.42		13.77		5.09	3.89
Rhineland-Palatine, Germany	Estimated (Raw/4)		0.12	0.85		2.25		3.61		3.44		1.27	0.97
		40	45	50	55	60	65	70	75	80	85	90	
Kapetanakis et al. 2016 [5]	Total	0.4	0.5	0.7	1.0	1.4	2.0	2.7	3.8	5.3	7.3	10.0	
White, selected ages, estimations	CI	0.2–0.5	0.4–0.7	0.5–1.0	0.7–1.4	1.0–1.9	1.5–2.7	2.1–3.7	2.9–5.1	4.0–7.1	5.5–9.8	7.4–13.5	
by meta-analysis	Men	0.4	0.6	0.8	1.1	1.5	2.0	2.8	3.8	5.1	7.0	9.4	
	CI	0.2–1.0	0.2–1.4	0.3–1.9	0.4–2.6	0.6–3.6	0.8–4.9	1.1–6.6	1.6–8.9	2.1–12.0	2.9–15.9	3.9–21.0	
	Women	0.4	0.5	0.6	0.9	1.2	1.6	2.1	2.8	3.7	4.9	6.5	
	CI	0.1–0.9	0.2–1.2	0.3–1.6	0.3–2.1	0.5–2.8	0.6–3.8	0.8–5.0	1.1–6.6	1.5–8.7	2.0–11.5	2.7–15.0	

However, two studies [8, 17] showed a decrease in the prevalence at the oldest age groups (80+ and 90+). The variation and differences between the number of rates may be explained by analyzing different data sources (e.g. administrative data or epidemiological surveys) but also by using different definitions of POAG [12].

While the number of studies that investigated the prevalence of POAG was limited, the number of studies on the incidence of POAG was even more restricted; there were only two (Table 2). By using data from the visual impairment project in Melbourne, Australia, Mukesh et al. estimated incidence rates by age groups, sex, and by varying level of validity of the POAG diagnosis [18]. They reported an average annual incidence of 0.10 per 100 person-years in total, 0.14 in males, and 0.06 in females at ages 40 and above [18]. The study of Cedrone et al. reported an increase of incidence rates from 0.07 at age 40–49 to 0.56 at age 70+. The total annual incidence was 0.32 [19]. The 5-year risk of definite POAG rate in the Rotterdam study was 0.6 (0.12 per year), with increasing rates of 1 for people aged 60 years to 3 for those aged 80 years (not shown) [20].

**Table 2:** Incidence rates from selected studies, transformed into average annual rates

Incidence	Years	Period		40–49	50–59	60–69	70–79	80+
Mukesh et al. 2002 [18] Melbourne, Australia Only definite POAG+	5	1992/94–1997-99	Total	0	0.02	0.12	0.28	0.82
			CI		0–0.08	0.02–0.22	0.12–0.44	0–1.82
			Men	0	0.06	0.2	0.4	0.8
			CI		0–0.16	0–0.4	0.1–0.7	0–2.22
			Women	0	0	0.06	0.14	0.82
			CI			0–0.18	0–0.42	0–2.28
				40–49	50–59	60–69	70+	
Cedrone et al. 2012 [19] Ponza, Italy	12	1988–2000	Total PAOG	0.07 0.01-	0.33	0.52	0.56	
			CI	0.38	0.15–0.78	0.15–0.88	0.2–2.26	

In the following, a summary of published risk factors is shown. A series of disease-related risk factors was identified in the literature (based on systematic literature reviews [1, 21]). Myopia [22–29] is found to increase the risk of glaucoma diseases, as well as hypertension, vasospasm, hypotension, and retinal vascular occlusion [30–34]. Increased risk of glaucoma was also reported for migraine [35, 36], injuries of the eye, and the orbit and degeneration of the iris and the ciliary body [37, 38], sleep apnea [39], and diabetes mellitus [34, 40, 41]. Contradictory and inconsistent results are found for smoking [42–46]. Sex does not appear to be a risk factor of POAG incidence based on the findings of Mukesh et al. and Cedrone et al. [18, 47]

## Methods

### Data

A proportionate age stratified random sample of 250,000 persons of all persons insured with the Allgemeine Ortskrankenkasse (AOK), the largest German public health insurer, was drawn. In 2018, about 32% of the German population was insured with the AOK. This proportion is stable at all ages, but increases to 47% in females at age 85 + [48].

All insured persons living in private households and nursing homes who were born in or before 1960 were tracked from 2010 through the end of 2013. The quarterly data cover general demographic information about sex and age, inpatient and outpatient ICD-10 diagnoses, medical treatments, and medication. Early withdrawal from the study was only possible due to a change in the insurer or to death. All diagnoses of treated diseases were recorded and reported by the physicians in hospitals and medical practices. The reported diagnoses were used as the calculation bases for the financial transfers from the health insurance to the physicians and hospitals.

POAG was defined using the ICD-10 classification H-40.1 stemming from outpatient (medical practices) and inpatient (discharge from hospital) diagnoses.

### Analysis samples and validation strategy

**Fig. 1:**
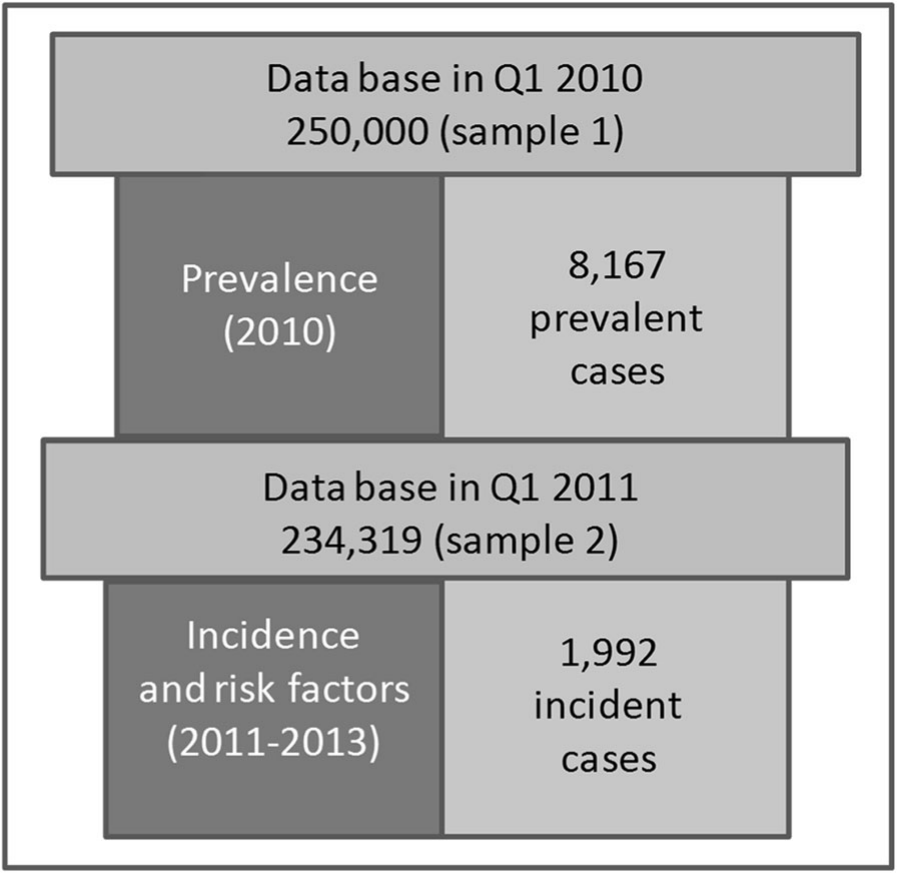
Flowchart of sample selection procedure for validated diagnoses, AOK data

Two analysis samples were constructed. For the estimation of age-specific prevalence sample 1 covered all 250,000 persons over the course of the four quarters of the year 2010. For the analysis of incidence sample 2 comprised all persons who did not have a valid POAG diagnosis in 2010 and were present at the beginning of 2011 (Fig. 1). Whether a POAG diagnosis was valid or not was decided by an internal validation strategy based on Schulz & Doblhammer [49] with the aim of increasing the sensitivity and decreasing the number of false-positive diagnoses of POAG. The strategy comprised two steps: In the first step diagnoses classified as “under suspicion” were not considered to be valid diagnoses. These are predominantly persons diagnosed with ocular hypertension but not with POAG. In the second step a validated diagnosis required at least two POAG diagnoses: one POAG diagnosis in one quarter and a minimum of one additional diagnosis by an ophthalmologist in any of the subsequent quarters of the observation period. If a patient died prior to the second diagnosis, he/she was not considered to be a prevalent or incident case. Non-ophthalmological diagnoses of POAG were not considered to be a valid diagnosis if there was no diagnosis made by an ophthalmologist in the same quarter. The size of sample 2 was 234,319 persons.

### Sensitivity analyses

In a first sensitivity analysis the effect of the validation strategy on the results was analyzed by modifying step 2 of the validation strategy insofar as only one diagnosis made by an ophthalmologist was required. This resulted sample 2 being reduced by 905 persons (*n* = 233,414) because more cases were identified as prevalent and thus excluded.

A second sensitivity analysis was conducted to explore whether the effects of the risk factors differed when persons ever diagnosed with diabetes were excluded. Thus, sample 2 was reduced by 67,426 persons (*n* = 166,893 persons). In a third sensitivity analysis we investigated the role of misclassification of the type of glaucoma by excluding all patients ever (prior or later) diagnosed with another type of glaucoma (angle-closure glaucoma, secondary glaucoma) than POAG. Sample 2 was reduced to 231,764 persons (minus 2555 persons).

### Control variables

In a multivariable analysis, we investigated potential predictors of POAG incidence. In addition to age and sex, we selected the following diseases: injuries of an eye and orbit (ICD-10-code S05), myopia (H44.2, H52.1, H52.5), retinal vascular occlusion (H34), papilledemia (H47.1), degeneration of iris and ciliary body (H21.2), migraine (G43), sleep apnea (G47.3), vasospasm (I73.9), diabetes mellitus type 1 and type 2 (E10-E14), hypertension (I10-I15), hypotension (I95), ischemic heart disease (I20-I25), and obesity (E65-E68). Indirect indicators for smoking are diagnoses of smoking related types of cancer (C00-C06, C10-C13, C15, C16, C18, C19, C20, C21, C25, C30-C34, C53-C56, C64, C67, C92, D09.0) and of obstructive pulmonary diseases (J40-J44, J47).

Diseases were validated by the validation strategy described above but were not restricted to ophthalmologists and coded as “ever” variables with the value one from the first valid diagnosis onward and zero otherwise. To ensure that the selected health problems were predictors and not coincidences, those diseases diagnosed within the same quarter as the incidence of POAG were coded as not causal factors (zero). The selected diseases had to have been diagnosed after the first quarter of 2010 and prior to the first valid POAG diagnosis.

## Methods

The **period prevalence** for 2010 was defined as the proportion of persons with a POAG diagnosis in at least one of the quarters of the year, divided by the number of all insured persons in the first quarter of 2010 (Equ. 1).

1 $$ {\mathrm{Period}\ \mathrm{prevalence}}_{2010,\mathrm{x},\mathrm{a}}=\frac{{\mathrm{Pre}}_{\mathrm{Q}1,2010,\mathrm{x},\mathrm{a}}+{\mathrm{Inc}}_{\mathrm{Q}2,2010,\mathrm{x},\mathrm{a}}+{\mathrm{Inc}}_{\mathrm{Q}3,2010,\mathrm{x},\mathrm{a}}+{\mathrm{Inc}}_{\mathrm{Q}4,2010,\mathrm{x},\mathrm{a}}}{{\mathrm{Pop}}_{\mathrm{Q}1,2010,\mathrm{x},\mathrm{a}}}\bullet 100 $$

Pre_Q1 2010, x, a_ was the sex (x)- and age group (a)-specific number of POAG patients (Pre) in the first quarter of 2010, Inc. were new POAG patients (incidence cases) in the following quarters Q2, Q3, and Q4 with identical sex and within the same age group, and Pop_Q1, 2010, x, a_ was the population in the first quarter of 2010. In the following, period prevalence is abbreviated as prevalence.

**Incidence** was specified as the first occurrence of a POAG diagnosis, which was considered an irreversible event which can be experienced only once in the lifetime of each person. POAG diagnosed persons in 2010 were excluded and exclusively new incident cases in 2011–2013 were analyzed (Sample 2). For all persons included, person-time under risk started with the first quarter of 2011 and ended with a POAG diagnosis in one of the following quarters or the exit of a person due to change of the insurer or death. Because the validation strategy required by definition at least two successive quarters in order to generate incident cases, the last two quarters were excluded from the calculation of the incidence.^1^ The incidence rate is the sex (x)- and age group (a)-specific number of persons with a first POAG diagnosis in 2011 to the middle of 2013 (Inc_2011 − 13, x, a_), divided by the person-time (person-years) under risk (PY_Risk_) from 2011 to the middle of 2013 (Equ. 2).

2 $$ {\mathrm{Inc}\mathrm{idence}}_{2011-13,\mathrm{x},\mathrm{a}}=\frac{{\mathrm{Inc}}_{2011-13,\mathrm{x},\mathrm{a}}}{{\mathrm{PY}}_{\mathrm{Risk},2011-13,\mathrm{x},\mathrm{a}}}\bullet 100 $$

Prevalence and incidence rates were calculated by age at the time of diagnosis for 5-year age groups (50–54, 55–59, 60–64, 65–69, 70–74, 75–79, 80–84, 85–89, 90+) and sex. 95% binomial exact confidence intervals were computed. Direct age standardization of the prevalence and incidence rates used the sex- and age-stratified 2010 German population. Differences in the age-specific prevalence and incidence between the sexes were tested using Pearson’s chi-squared tests.

A **Cox proportional hazard model** was used to estimate the simultaneous influence of major risk factors on the incidence of POAG in 2011–2013. All analyses were performed using Stata version 12.1.

## Results

### Descriptives of the analysis populations

In the first quarter of 2010, 55.8% of sample 1 was female, and the sample mean age was 65.9 years (SD: 11.8). The mean age of females was more than 3 years higher than that of males (67.5 years versus 63.9 years).

In sample 2 we observed 234,319 persons and the mean age of the population in the first quarter of 2010 was 66.2 (SD: 11.6) years, 55.6% of the sample was female, the mean age difference between the two sexes was more than 3 years (67.7 versus 64.2) (Table 3).

**Table 3:** Descriptive overview of the two samples, AOK data

		Sample 1 2010	Sample 2 2011–2013
Total mean age (SD)	at the beginning of the first quarter of the starting year	65.9 (11.8)	66.2 (11.6)
Male mean age (SD)		63.9 (10.8)	64.2 (10.6)
Female mean age (SD)		67.5 (12.3)	67.7 (12.1)
% male		44.3	44.4
% female		55.7	55.6

In sample 2, which was used for the analysis of POAG risk factors, the most frequent diagnoses in the first quarter of 2011 were hypertension (28.83%), diabetes mellitus (23.69%), ischemic heart disease (17.98%), obesity (15.11%), obstructive pulmonary disease (14.11%), and myopia (5.15%). Papilledema (0.03%), degeneration of the iris and the ciliary body (0.04%), injuries of eye and orbit (0.09%), and retinal vascular occlusions (0.37%) were rarely diagnosed (Table 4).

**Table 4:** Descriptive overview of sample 2, first quarter in 2011, AOK data

Factors		Persons	Proportion
Age	50–54	51,332	21.91%
	55–59	33,320	14.22%
	60–64	27,292	11.65%
	65–69	26,457	11.29%
	70–74	34,172	14.58%
	75–79	26,292	11.22%
	80–84	19,468	8.31%
	85–89	11,213	4.79%
	90+	4773	2.04%
Sex	Males	104.134	44.44%
	Females	130,185	55.56%
Comorbidities	Ever hypertension	67,549	28.83%
	Ever diabetes mellitus	55,499	23.69%
	Ever ischemic heart disease	42,128	17.98%
	Ever obesity	35,401	15.11%
	Ever obstructive pulmonary disease	33,065	14.11%
	Ever myopia	12,059	5.15%
	Ever vasospasm	9558	4.08%
	Ever smoking related cancer	8215	3.51%
	Ever migraine	6813	2.91%
	Ever sleep apnea	4830	2.06%
	Ever hypotension	4372	1.87%
	Ever retinal vascular occlusions	868	0.37%
	Ever injury of eye and orbit	221	0.09%
	Ever degeneration of iris and ciliary body	99	0.04%
	Ever papilledema	68	0.03%

### Prevalence

A total of 8167 persons were defined as POAG patients, which resulted in an age-standardized prevalence of 3.22% [95%-CI: 3.15–3.29%] for the total population; 2.90% [95%-CI: 2.80–3.00%, 3132 persons] for males and 3.49% [95%-CI: 3.40–3.59%, 5035 persons] for females.

**Fig. 2:**
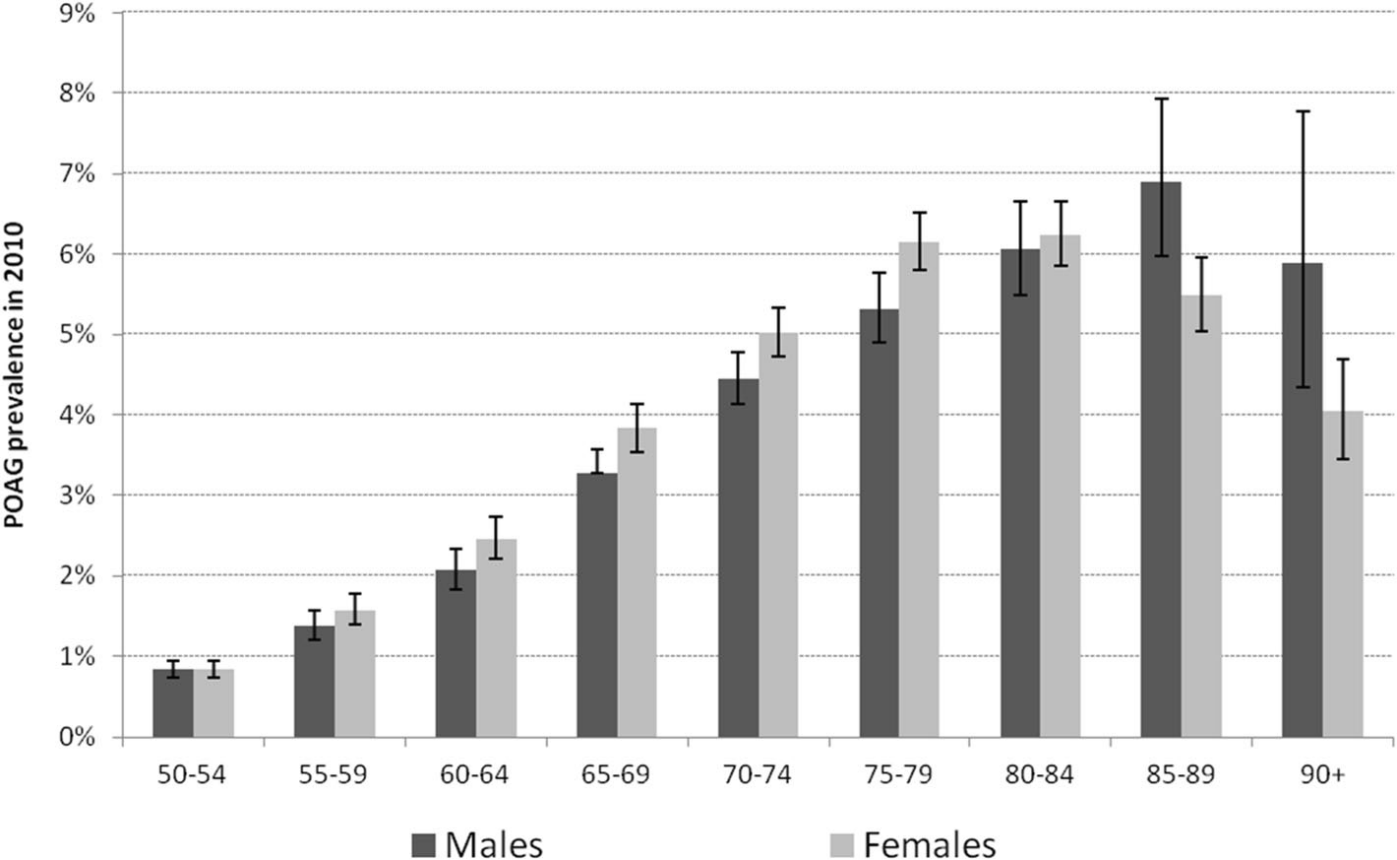
POAG prevalence by age and sex, including 95%-CI, 2010, AOK data

In case of the age-specific prevalence, the figures showed a similar pattern for both sexes (Fig 2). The age-specific prevalence increased more than seven-fold up to age 80–84 in females and age 85–89 in males and decreased thereafter (Table 5). The increase was slightly steeper for females than for males, which was also true for the decrease in prevalence at the highest ages. The POAG prevalence of females was significantly higher at ages 60–64 (Pearson chi [2]=4.51, *p* = 0.034), 65–69 (6.41, *p* = 0.011), 70–74 (5.97, *p* = 0.015), and 75–79 (7.36, *p* = 0.007), while it was significantly lower at ages 85–89 (6.81, *p* = 0.009) and 90+ (4.90, *p* = 0.027).

**Table 5:** POAG prevalence by age and sex, 2010, AOK data

	Age	Prevalence (%), 95%-CI (Binomial Exact)		Prevalent persons		Persons on 01.01.2010
Males	50–54	0.84	(0.74–0.95)^1^	252	8.0%	29,949
	55–59	1.38	(1.21–1.57)	227	7.2%	16,418
	60–64	2.07	(1.83–2.33)	271	8.7%	13,090
	65–69	3.27	(2.99–3.57)	473	15.1%	14,465
	70–74	4.44	(4.13–4.77)	715	22.8%	16,102
	75–79	5.32	(4.90–5.76)	562	17.9%	10,568
	80–84	6.06	(5.49–6.66)	400	12.8%	6605
	85–89	6.91	(5.98–7.93)	186	5.9%	2693
	90+	5.89	(4.34–7.78)	46	1.5%	781
	Total			3132		110,671
Females	50–54	0.84	(0.73–0.95)	242	4.8%	28,932
	55–59	1.58	(1.39–1.78)	264	5.2%	16,739
	60–64	2.47	(2.21–2.74)	334	6.6%	13,538
	65–69	3.83	(3.54–4.14)	614	12.2%	16,042
	70–74	5.02	(4.72–5.33)	1007	20.0%	20,070
	75–79	6.15	(5.80–6.52)	1043	20.7%	16,953
	80–84	6.25	(5.85–6.66)	860	17.1%	13,770
	85–89	5.49	(5.03–5.97)	510	10.1%	9296
	90+	4.04	(3.45–4.69)	161	3.2%	3989
	Total			5035		139,329
Total	50–54	0.84	(0.77–0.92)	494	6.0%	58,881
	55–59	1.48	(1.35–1.62)	491	6.0%	33,157
	60–64	2.27	(2.10–2.46)	605	7.4%	26,628
	65–69	3.56	(3.36–3.78)	1087	13.3%	30,507
	70–74	4.76	(4.54–4.99)	1722	21.1%	36,172
	75–79	5.83	(5.56–6.12)	1605	19.7%	27,521
	80–84	6.18	(5.86–6.52)	1260	15.4%	20,375
	85–89	5.81	(5.39–6.24)	696	8.5%	11,989
	90+	4.34	(3.78–4.96)	207	2.5%	4770
	Total			8167		250,000

### Incidence

The age-standardized POAG incidence rate was significantly higher for woman than for males. Age-standardized incidence was 0.38 (95%-CI: 0.36–0.39, 1992 persons) persons per 100 person-years, the male incidence was 0.32 (95%-CI: 0.29–0.34; 735 persons) and the female was 0.43 (95%-CI: 0.40–0.45; 1257 persons).

**Fig. 3:**
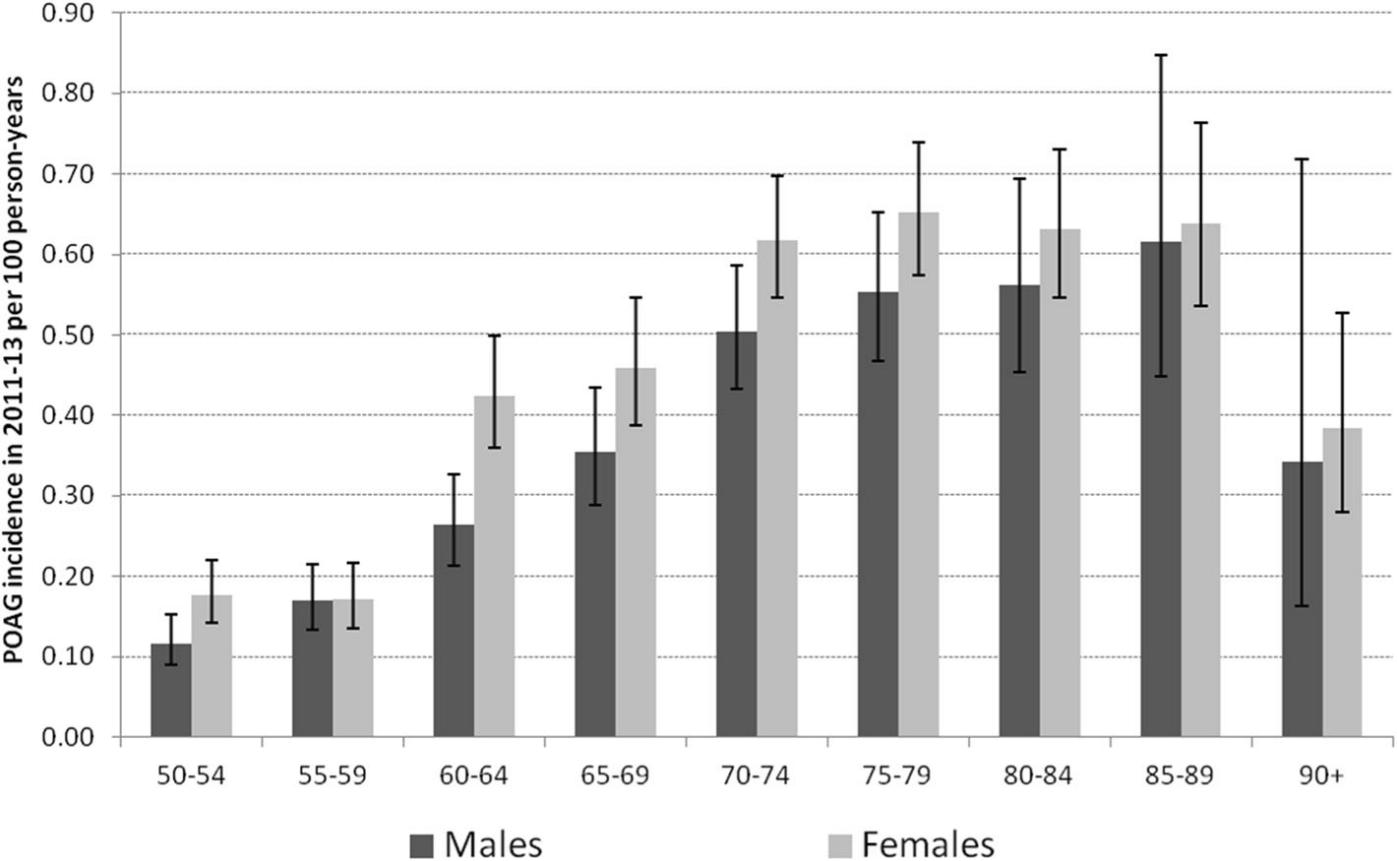
POAG incidence rate by age and sex, including 95%-CI, 2011–2013, AOK data

Considering the age-specific incidence rates, they increased continuously until age 85–89 and decreased at the highest ages (Fig 3). The age pattern was similar for both sexes; however, the incidence was significantly higher for females than for males at the ages 60–64 (Table 6).

**Table 6:** POAG incidence rate by age and sex, 2011–2013, AOK data

	Age	Incidence rate (per 100 person-years), 95%-CI (Binomial Exact)	Incident persons		Person-years
Males	50–54	0.12 (0.09–0.15)	54	7.3%	46,459
	55–59	0.17 (0.13–0.22)	68	9.3%	40,016
	60–64	0.26 (0.21–0.33)	85	11.6%	32,217
	65–69	0.35 (0.29–0.43)	92	12.5%	26,018
	70–74	0.50 (0.43–0.59)	169	23.0%	33,551
	75–79	0.55 (0.47–0.65)	137	18.6%	24,803
	80–84	0.56 (0.45–0.69)	85	11.6%	15,156
	85–89	0.62 (0.45–0.85)	38	5.2%	6164
	90+	0.34 (0.16–0.72)	7	1.0%	2042
	Total		735		226,425
Females	50–54	0.18 (0.14–0.22)	80	6.4%	45,100
	55–59	0.17 (0.14–0.22)	69	5.5%	40,181
	60–64	0.42 (0.36–0.50)	143	11.4%	33,707
	65–69	0.46 (0.39–0.55)	130	10.3%	28,303
	70–74	0.62 (0.55–0.70)	251	20.0%	40,674
	75–79	0.65 (0.57–0.74)	240	19.1%	36,820
	80–84	0.63 (0.55–0.73)	184	14.6%	29,120
	85–89	0.64 (0.54–0.76)	122	9.7%	19,087
	90+	0.38 (0.28–0.53)	38	3.0%	9901
	Total		1257		282,893
Total	50–54	0.15 (0.12–0.17)	134	6.7%	91,559
	55–59	0.17 (0.14–0.20)	137	6.9%	80,198
	60–64	0.35 (0.30–0.39)	228	11.4%	65,924
	65–69	0.41 (0.36–0.47)	222	11.1%	54,321
	70–74	0.57 (0.51–0.62)	420	21.1%	74,225
	75–79	0.61 (0.55–0.68)	377	18.9%	61,623
	80–84	0.61 (0.54–0.68)	269	13.5%	44,275
	85–89	0.63 (0.54–0.74)	160	8.0%	25,251
	90+	0.38 (0.28–0.50)	45	2.3%	11,943
	Total		1992		509,318

### Multivariable results

The multivariable Cox-regression model and hazard ratios (HR) showed a curvilinear age effect with an increase up to age 75–79 (HR = 3.67 [95%-CI: 3.04–4.44] as compared to age 50–54) and a decrease at the highest ages (HR = 2.39 [95%-CI: 1.74–3.27]) (Table 7). Females experienced a significantly increased risk compared to males, with HR = 1.19 [95%-CI: 1.09–1.30]. The most important predictors of POAG incidence were the earlier presence of an injury of the eye and orbit with HR = 2.75 [95%-CI: 1.56–4.86], myopia with HR = 2.55 [95%-CI: 2.29–2.84], and the degeneration of iris and ciliary body with HR = 2.55 [95%-CI: 1.14–5.70]. Retinal vascular occlusions had a HR of 2.34 [95%-CI: 1.71–3.20], diabetes mellitus Type 1 or Type 2 had a HR of 1.23 [95%-CI: 1.13–1.35], and hypertension had a HR of 1.13 [95%-CI: 1.03–1.24]. In contrast, smoking-related types of cancer had a lower incidence of POAG with a HR of 0.78 [95%-CI: 0.63–0.96].

**Table 7:** Results of the Cox-regression model, risk of incidence of POAG, 2011–2013, AOK data

	Factors	Hazard Ratio	*p*-Value	95%-Confidence
Age	50–54	1		
	55–59	1.25	0.045	(1.00–1.55)
	60–64	2.20	< 0.001	(1.80–2.69)
	65–69	2.49	< 0.001	(2.04–3.05)
	70–74	3.37	< 0.001	(2.80–4.06)
	75–79	3.67	< 0.001	(3.04–4.44)
	80–84	3.49	< 0.001	(2.85–4.26)
	85–89	3.44	< 0.001	(2.75–4.31)
	90+	2.39	< 0.001	(1.74–3.27)
Sex	Women (Ref. Men)	1.19	< 0.001	(1.09–1.30)
Morbidities (selection)	Ever degeneration of iris and ciliary body	2.55	0.22	(1.14–5.70)
	Ever myopia	2.55	<0.001	(2.29–2.84)
	Ever injury of eye and orbit	2.75	<0.001	(1.56–4.86)
	Ever retinal vascular occlusions	2.34	<0.001	(1.71–3.20)
	Ever diabetes mellitus	1.23	<0.001	(1.13–1.35)
	Ever hypertension	1.13	0.008	(1.03–1.24)
	Ever smoking related cancer	0.78	0.020	(0.63–0.96)

Note: Insignificant hazard ratios are not shown in the table, but the model is further adjusted for papilledema, hypotension, ischemic heart disease, migraine, sleep apnea, obstructive pulmonary disease, vasospasm and obesity

Papilledema (HR = 2.38, *p* = 0.134), hypotension (HR = 0.95, *p* = 0.705), ischemic heart disease (HR = 1.06, *p* = 0.228), migraine (HR = 1.15, *p* = 0.239), sleep apnea (HR = 1.22, *p* = 0.087), obstructive pulmonary disease (HR = 0.98, *p* = 0.722), vasospasm (HR = 1.01, *p* = 0.891), and obesity (HR = 1.01, *p* = 0.818) were not significantly linked to the POAG incidence when controlled for other diseases (not shown in Table 7).

### Sensitivity analyses

We performed three sensitivity analyses: The first evaluated the consequences of the validation strategy on prevalence, incidence rate, and the effects of the risk factors. Using the alternative validation strategy, a significantly higher age-standardized prevalence was observed (3.60% [95%-CI: 3.53–3.67%, 9141 persons] for the total population, 3.22% [95%-CI: 3.12–3.33%; 3481 persons] for males and 3.92% [95%-CI: 3.82–4.02%, 5660 persons] for females), while the age-specific prevalences (Additional file 1: Table S1) were higher at all ages, but did not differ significantly (Table 5). The age-standardized incidence rate was significantly higher (0.54 [95%-CI: 0.52–0.56; 2823 persons] for the total population; 0.47 [95%-CI: 0.44–0.50; 1078 persons] for males and 0.60 [95%-CI: 0.57–0.62; 1745 persons] for females). While age-specific incidence rates were higher in the sensitivity analysis, the differences between the two validation strategies were statistically not significant (Table 6 and Additional file 1: Table S2). Results of the multivariable models remained largely unchanged (see Additional file 1: Tables S3 and S4).

In a second sensitivity analysis, we reduced sample 2 by patients ever diagnosed with diabetes in the observation period, which led to nearly identical results (Additional file 1: Table S5).

In a third sensitivity analysis, we reduced sample 2 by patients prior or later diagnosed with angle-closure or secondary glaucoma in the observation period, and results remained largely unchanged (Additional file 1: Table S6).

## Discussion

In this study, age-standardized and age-specific prevalence and incidence rates of POAG were investigated for the population aged 50 and above. Our prevalence estimates (both sexes combined: 3.22%) were at the upper level compared to prevalences from previously published studies, but within the reported range (0.97% [17] and 3.54% [7]). This study’s incidence rate of about 0.38 per 100 person-years was higher than the range of 0.10 and 0.32 reported by Mukesh et al. and Cedrone et al. [18, 19]

The age-standardized prevalence and incidence were higher for females than for males. This higher incidence was confirmed in the multivariable analysis, in which we controlled for age and major disease-related risk factors. Mukesh et al. did not find any sex-specific differences in POAG incidence; however, the total number of incident persons in our study was much higher than the number of incident persons in their study [18].

Prevalence and incidence increased with age up to age 80 and declined thereafter. Comparing the sexes, both the prevalence and incidence were significantly higher for females than for males at ages less than 80, while there was no gender difference at the higher ages. The consistently higher prevalence and incidence of females may be explained by a higher risk of POAG, but also by additional factors. One explanation may be differences in health behavior, including health awareness, health seeking, health care utilization, and adherence to therapies. The higher health awareness of females may cause more health seeking behavior and health care utilization than males [50]. Thus, POAG may be detected at earlier ages in females than in males. At the highest ages, gender disparities in health care utilization may diminish due to large multi-morbidity present among both genders. Because claims data do not contain any information about socioeconomic status, it remains open whether these gender differences were the result of a selection bias, with comparatively more women than men with low socioeconomic status being insured with the AOK.

The general decline of the prevalence and the incidence at the highest ages may be the result of a series of factors, such as a decrease in health care utilization, an increase in competing health risks (e.g. other eye diseases or life-threatening diseases), and the effect of mortality selection. The effect of mortality selection is also called “cohort inversion” [51] or “unobserved heterogeneity in combination with mortality selection” [52]. It describes the phenomenon of decreasing disparities in health at the highest ages. This finding can be explained by a trend of decreasing heterogeneity in health and life style due to the deaths of persons with risky life styles and poor health, while the fitter persons with a generally lower risk of morbidities, among them POAG and its risk factors, reach the highest ages.

The longitudinal design of the study permitted the investigation of risk factors of POAG incidence. One of the many strengths of the dataset was the wide range of diagnoses from all fields of medicine. The multivariable regression model confirmed the positive associations of POAG with myopia [22–28] and diabetes [34, 40, 41], as well as with injuries of the eye and the orbit, degenerations of iris and ciliary body, retinal vascular occlusions, and hypertension [31, 32].

With these factors, we found some unexpected associations, not yet discussed in the literature in connection with POAG. At some stage of our investigation, we expected that misdiagnosed secondary glaucoma would influence our results. Therefore, all patients diagnosed with secondary glaucoma were excluded in a second sensitivity analysis from the overall cohort (see section sensitivity analyses). Still, the same correlation was found, showing a clear association of injuries of the eye and the orbit, degenerations of iris and ciliary body, retinal vascular occlusions and hypertension as risk factors of POAG.

A 23% higher link to POAG was found for diabetes mellitus, which was consistent with some earlier studies [34, 40, 41, 53]. In Germany, a public health program for diabetes (Diabetes Disease Management Program) exists which aims to coordinate therapies and to increase cooperation of general practitioners and ophthalmologists. Due to this program and the characteristics of claims data the higher incidence in patients with diabetes may also be the result of an increased utilization of ophthalmic services which may lead to a more frequent diagnosis of POAG. However, sensitivity analysis showed that the results were nearly identical after the exclusion of patients ever diagnosed with diabetes in the observation period (Additional file 1: Table S5).

The positive effect for smoking-related cancers was consistent with the findings from Buys et al. [46] but was in contrast to Jain et al. [45] Because claims data do not contain information about smoking status, comparisons with the findings from the literature are limited. The protective effect of nicotine might be explained by its effect on cerebral blood flow, which is increased by nicotine and therefore possibly leads to an increased oxygen consumption of the brain. The optic nerve as a part of the central nervous system might experience the same positive effects by nicotine [43, 55].

Our study has a series of strengths. An important strength is that POAG diagnoses were based on evaluations by ophthalmologists, which assure solid validity, as the German statutory health insurance system implements visits to ophthalmologists every 3 month when suffering from a chronic disease without any extra costs for patients. We did sensitivity analyses to investigate the role of misclassification of the type of glaucoma (open-angle glaucoma, angle-closure glaucoma, secondary glaucoma). By excluding all patients ever (prior or later) diagnosed with another type of glaucoma than POAG, the models showed very robust results (Additional file 1: Table S6). Thus, problems of misclassification of the type of glaucoma are assumed to be marginal.

The large random sample drawn from an official process-generated data source did not suffer from self-selected dropouts due to unwillingness to participate in a study as is often the case in survey-based studies. The large sample size promotes the representativeness of the results, especially in studies about morbidities with a low prevalence. However, Hoffmann and Koller showed that the AOK members had a lower socioeconomic status than persons insured with other public insurance agencies [54]. This may explain the higher prevalence and incidence of our study. An additional advantage was that populations in private households and nursing homes were covered, while the latter are usually absent from survey-based studies. The choice of the comorbidities may be discussed, as some of the diseases may be simply coincident morbidities than causes of glaucoma. This problem was reduced by considering the chronology of the diagnoses of POAG and the particular comorbidities.

However, there were also some limitations to our work. When comparing claims data from different countries, there is no common standardized definition with defined inclusion criteria of a POAG diagnosis. Using different criteria and definitions leads to different prevalence estimates and POAG numbers [5], yet this limitation is an overall problem common to this field, as the criteria for glaucoma diagnoses are also not standardized [12]. While some studies used indirect methods, such as the combination of symptoms of glaucoma diseases or self-reported visual impairments, or included intraocular pressure [5], our study used ICD diagnoses issued by ophthalmologists. We assumed a high validity of the diagnosis; however, claims data aim to document medical treatments and their costs rather than disease processes. We were not able to identify false-negative diagnoses which may result in an underestimation of POAG occurrence. False-positive diagnoses, however, were reduced by the internal validation strategy. Results changed only marginally when an alternative validation strategy was applied, indicating a marginal influence of false-positive POAG diagnoses. Furthermore, the methodological definition of prevalence affected the comparability. In this study, period prevalence was used, whereas in most other studies, it is not clearly stated whether period or point prevalence was used. Another limitation is the fact that only diseases which were documented and reported by using ICD codes were covered in the analysis. Risk factors like physical activity, diet, alcohol and tobacco consumption were not available. Smoking as a risk factor was measured indirectly by severe diseases related to smoking, but covered predominately heavy (former and current) smokers.

## Conclusions

Our study has two major results: First, we presented estimates for the prevalence and incidence of POAG in Germany, based on a large data set and compared these with earlier studies.

Second, longitudinal data with large numbers of incident cases were used to investigate sex disparities, age structure, and risk factors of POAG. We were able to show a significantly higher incidence rate and prevalence for females compared to males, an increasing POAG incidence and prevalence with increasing age, and influential risk factors such as injuries of the eye and orbit, degenerations of iris and ciliary body, myopia, retinal vascular occlusions, and diabetes.

It is of immense importance for quality of life of the individual patient to understand and reduce the burden of this disease. Our findings identified risk factors of POAG which may have severe consequences on its onset and course. Therefore, our results may serve as a base to develop intervention measures, which may increase the awareness of patients and physicians and thus reduce the incidence of POAG in the future.

## Additional file

Additional file 1: **Table S1.** Prevalence by age and sex, alternative validation strategy, 2010, AOK data. **Table S2.** Incidence rate by age and sex, alternative validation strategy, 2011–2013, AOK data. **Table S3.** Results of the complete Cox-regression model, risk of incidence of POAG, 2011-2013, AOK data. **Table S4.** Results of the complete Cox-regression model, risk of incidence of POAG, alternative validation strategy, 2011-2013, AOK data. **Table S5**. Results of the complete Cox-regression model, risk of incidence of POAG, persons with diabetes excluded, 2011–2013, AOK data. **Table S6.** Results of the complete Cox-regression model, risk of incidence of POAG, persons with (prior or later) primary angle-closure glaucoma (H40.2) and secondary glaucoma (H40.3–6) excluded, 2011–2013, AOK data. (DOCX 1680 kb)

## Abbreviations

AOK: Allgemeine Ortskrankenkassen
CI: Confidence interval
HR: Hazard ratio
ICD: International classification of diseases
POAG: Primary open-angle glaucoma
SD: Standard deviation

## Footnotes

1 In the validation strategy the last two quarters of 2013 were excluded for not underestimating incidence rates, as the number of valid incidence cases was too low.
