# Supplementary material for: Prevalence, incidence, and risk factors of primary open-angle glaucoma - a cohort study based on longitudinal data from a German public health insurance
Source: BMC Public Health. 2019 Jul 1;19:851. doi: 10.1186/s12889-019-6935-6 (PMC6604230; doi:10.1186/s12889-019-6935-6)
Supplement: Supplementary file 1 — Table S1. Prevalence by age and sex, alternative validation strategy, 2010, AOK data. Table S2. Incidence rate by age and sex, alternative validation strategy, 2011–2013, AOK data. Table S3. Results of the complete Cox-regression model, risk of incidence of POAG, 2011-2013, AOK data. Table S4. Results of the complete Cox-regression model, risk of incidence of POAG, alternative validation strategy, 2011-2013, AOK data. Table S5. Results of the complete Cox-regression model, risk of incidence of POAG, persons with diabetes excluded, 2011–2013, AOK data. Table S6. Results of the complete Cox-regression model, risk of incidence of POAG, persons with (prior or later) primary angle-closure glaucoma (H40.2) and secondary glaucoma (H40.3–6) excluded, 2011–2013, AOK data. (DOCX 1680 kb) [file 12889_2019_6935_MOESM1_ESM.docx]

**Supplementary Material**

**Table S1**

|  | **Age** | **Prevalence (%),  95%-CI (Binomial Exact)** | | | | | | **Prevalent persons** | |  | **Persons on 01.01.2010** |
| --- | --- | --- | --- | --- | --- | --- | --- | --- | --- | --- | --- |
| **Males** | 50-54 | **0.97** | ( | *0.86* | *-* | *1.09* | ) | 290 | 8.3% |  | 29,949 |
|  | 55-59 | **1.58** | ( | *1.40* | *-* | *1.79* | ) | 260 | 7.5% |  | 16,418 |
|  | 60-64 | **2.28** | ( | *2.04* | *-* | *2.55* | ) | 299 | 8.6% |  | 13,090 |
|  | 65-69 | **3.58** | ( | *3.28* | *-* | *3.90* | ) | 518 | 14.9% |  | 14,465 |
|  | 70-74 | **4.76** | ( | *4.44* | *-* | *5.10* | ) | 767 | 22.0% |  | 16,102 |
|  | 75-79 | **5.85** | ( | *5.41* | *-* | *6.31* | ) | 618 | 17.8% |  | 10,568 |
|  | 80-84 | **6.92** | ( | *6.32* | *-* | *7.56* | ) | 457 | 13.1% |  | 6,605 |
|  | 85-89 | **7.87** | ( | *6.88* | *-* | *8.95* | ) | 212 | 6.1% |  | 2,693 |
|  | 90+ | **7.68** | ( | *5.91* | *-* | *9.78* | ) | 60 | 1.7% |  | 781 |
|  | **Total** |  |  |  |  |  |  | 3,481 | |  | 110,671 |
|  |  |  |  |  |  |  |  |  |  |  |  |
|  | **Age** | **Prevalence (%),  95%-CI (Binomial Exact)** | | | | | | **Prevalent persons** | |  | **Persons on 01.01.2010** |
| **Females** | 50-54 | **1.00** | ( | *0.89* | *-* | *1.12* | ) | 289 | 5.1% |  | 28,932 |
|  | 55-59 | **1.77** | ( | *1.58* | *-* | *1.99* | ) | 297 | 5.2% |  | 16,739 |
|  | 60-64 | **2.79** | ( | *2.52* | *-* | *3.08* | ) | 378 | 6.7% |  | 13,538 |
|  | 65-69 | **4.31** | ( | *4.00* | *-* | *4.64* | ) | 692 | 12.2% |  | 16,042 |
|  | 70-74 | **5.49** | ( | *5.18* | *-* | *5.81* | ) | 1,102 | 19.5% |  | 20,070 |
|  | 75-79 | **6.78** | ( | *6.40* | *-* | *7.17* | ) | 1,149 | 20.3% |  | 16,953 |
|  | 80-84 | **7.03** | ( | *6.61* | *-* | *7.47* | ) | 968 | 17.1% |  | 13,770 |
|  | 85-89 | **6.40** | ( | *5.91* | *-* | *6.92* | ) | 595 | 10.5% |  | 9,296 |
|  | 90+ | **4.76** | ( | *4.12* | *-* | *5.47* | ) | 190 | 3.4% |  | 3,989 |
|  | **Total** |  |  |  |  |  |  | 5,660 | |  | 139,329 |
|  |  |  |  |  |  |  |  |  |  |  |  |
|  | **Age** | **Prevalence (%),  95%-CI (Binomial Exact)** | | | | | | **Prevalent persons** | |  | **Persons on 01.01.2010** |
| **Total** | 50-54 | **0.98** | ( | *0.91* | *-* | *1.07* | ) | 579 | 6.3% |  | 58,881 |
|  | 55-59 | **1.68** | ( | *1.54* | *-* | *1.82* | ) | 557 | 6.1% |  | 33,157 |
|  | 60-64 | **2.54** | ( | *2.36* | *-* | *2.74* | ) | 677 | 7.4% |  | 26,628 |
|  | 65-69 | **3.97** | ( | *3.75* | *-* | *4.19* | ) | 1,210 | 13.2% |  | 30,507 |
|  | 70-74 | **5.17** | ( | *4.94* | *-* | *5.40* | ) | 1,869 | 20.4% |  | 36,172 |
|  | 75-79 | **6.42** | ( | *6.13* | *-* | *6.72* | ) | 1,767 | 19.3% |  | 27,521 |
|  | 80-84 | **6.99** | ( | *6.65* | *-* | *7.35* | ) | 1,425 | 15.6% |  | 20,375 |
|  | 85-89 | **6.73** | ( | *6.29* | *-* | *7.19* | ) | 807 | 8.8% |  | 11,989 |
|  | 90+ | **5.24** | ( | *4.63* | *-* | *5.91* | ) | 250 | 2.7% |  | 4,770 |
|  | **Total** |  |  |  |  |  |  | 9,141 | |  | 250,000 |

**Table S2**

|  | **Age** | **Incidence rate  (per 100 person-years),  95%-CI (Binomial Exact)** | | | | | | **Incident persons** | |  | **Person-years** |  |
| --- | --- | --- | --- | --- | --- | --- | --- | --- | --- | --- | --- | --- |
| **Males** | 50'-54 | **0.21** | ( | *0.18* | *-* | *0.26* | ) | 99 | 9.2% |  | 46,367 |  |
|  | 55-59 | **0.23** | ( | *0.19* | *-* | *0.29* | ) | 93 | 8.6% |  | 39,927 |  |
|  | 60-64 | **0.40** | ( | *0.33* | *-* | *0.47* | ) | 127 | 11.8% |  | 32,107 |  |
|  | 65-69 | **0.52** | ( | *0.44* | *-* | *0.61* | ) | 134 | 12.4% |  | 25,913 |  |
|  | 70-74 | **0.71** | ( | *0.63* | *-* | *0.81* | ) | 238 | 22.1% |  | 33,396 |  |
|  | 75-79 | **0.82** | ( | *0.72* | *-* | *0.94* | ) | 202 | 18.7% |  | 24,609 |  |
|  | 80-84 | **0.78** | ( | *0.65* | *-* | *0.93* | ) | 117 | 10.9% |  | 15,012 |  |
|  | 85-89 | **0.90** | ( | *0.69* | *-* | *1.18* | ) | 55 | 5.1% |  | 6,091 |  |
|  | 90+ | **0.65** | ( | *0.38* | *-* | *1.11* | ) | 13 | 1.2% |  | 2,012 |  |
|  | **Total** |  |  |  |  |  |  | 1,078 | |  | 225,434 |  |
|  |  |  |  |  |  |  |  |  |  |  |  |  |
|  | **Age** | **Incidence rate  (per 100 person-years),  95%-CI (Binomial Exact)** | | | | | | **Incident persons** | |  | **Person-years** |  |
| **Females** | 50'-54 | **0.26** | ( | *0.22* | *-* | *0.31* | ) | 118 | 6.8% |  | 44,996 |  |
|  | 55-59 | **0.27** | ( | *0.22* | *-* | *0.33* | ) | 108 | 6.2% |  | 40,079 |  |
|  | 60-64 | **0.55** | ( | *0.47* | *-* | *0.63* | ) | 184 | 10.5% |  | 33,556 |  |
|  | 65-69 | **0.62** | ( | *0.54* | *-* | *0.72* | ) | 175 | 10.0% |  | 28,128 |  |
|  | 70-74 | **0.87** | ( | *0.79* | *-* | *0.97* | ) | 353 | 20.2% |  | 40,396 |  |
|  | 75-79 | **0.86** | ( | *0.77* | *-* | *0.96* | ) | 315 | 18.1% |  | 36,515 |  |
|  | 80-84 | **0.90** | ( | *0.80* | *-* | *1.02* | ) | 260 | 14.9% |  | 28,806 |  |
|  | 85-89 | **0.87** | ( | *0.75* | *-* | *1.02* | ) | 165 | 9.5% |  | 18,865 |  |
|  | 90+ | **0.68** | ( | *0.54* | *-* | *0.87* | ) | 67 | 3.8% |  | 9,787 |  |
|  | **Total** |  |  |  |  |  |  | 1,745 | |  | 281,131 |  |
|  |  |  |  |  |  |  |  |  |  |  |  |  |
|  | **Age** | **Incidence rate  (per 100 person-years),  95%-CI (Binomial Exact)** | | | | | | **Incident persons** | |  | **Person-years** |  |
| **Total** | 50'-54 | **0.24** | ( | *0.21* | *-* | *0.27* | ) | 217 | 7.7% |  | 91,363 |  |
|  | 55-59 | **0.25** | ( | *0.22* | *-* | *0.29* | ) | 201 | 7.1% |  | 80,007 |  |
|  | 60-64 | **0.47** | ( | *0.42* | *-* | *0.53* | ) | 311 | 11.0% |  | 65,663 |  |
|  | 65-69 | **0.57** | ( | *0.51* | *-* | *0.64* | ) | 309 | 10.9% |  | 54,041 |  |
|  | 70-74 | **0.80** | ( | *0.74* | *-* | *0.87* | ) | 591 | 20.9% |  | 73,792 |  |
|  | 75-79 | **0.85** | ( | *0.78* | *-* | *0.92* | ) | 517 | 18.3% |  | 61,125 |  |
|  | 80-84 | **0.86** | ( | *0.78* | *-* | *0.95* | ) | 377 | 13.4% |  | 43,819 |  |
|  | 85-89 | **0.88** | ( | *0.77* | *-* | *1.01* | ) | 220 | 7.8% |  | 24,956 |  |
|  | 90+ | **0.68** | ( | *0.54* | *-* | *0.84* | ) | 80 | 2.8% |  | 11,800 |  |
|  | **Total** |  |  |  |  |  |  | 2,823 | |  | 506,565 |  |

**Table S3**

**Table S4**

**Table S5**

**Table S6**
